# Gold Nanoparticles as a Possible Tool to Untangle Some Structural Features of the Gluten Network

**DOI:** 10.3390/foods14233985

**Published:** 2025-11-21

**Authors:** Davide Emide, Giovanni D’Auria, Stefania Iametti, Alberto Barbiroli, Mauro Marengo, Gianfranco Mamone, Pasquale Ferranti, Francesco Bonomi

**Affiliations:** 1 Department of Food, Environmental and Nutritional Sciences (DeFENS), Università degli Studi di Milano, Via G. Celoria 2, 20133 Milan, Italy; davide.emide@unimi.it (D.E.); alberto.barbiroli@unimi.it (A.B.); francesco.bonomi@unimi.it (F.B.); 2 Department of Agricultural Sciences, University of Naples Federico II, 80055 Portici, Italy; giovanni.dauria@unina.it (G.D.); pasquale.ferranti@unina.it (P.F.); 3 Department of Drug Science and Technology, University of Turin, 10125 Turin, Italy; mauro.marengo@unito.it; 4 Institute of Food Sciences, National Research Council, 83100 Avellino, Italy; gianfranco.mamone@isa.cnr.it

**Keywords:** gold nanoparticles, gluten network, cysteine thiols, thiol–disulfide exchange, durum wheat protein, protein aggregation, proteomics, cysteine accessibility, protein structure, size-based probes

## Abstract

Aqueous semolina suspensions were reacted with spherical gold nanoparticles (AuNPs, nominal diameter, 20 nm) to assess the accessibility of cysteine thiols in durum wheat proteins, focusing on network-forming gluten proteins. Unlike small thiol reagents, covalent bond formation between gold ions on the AuNPs surface and protein thiols was greatly facilitated by the addition of 1% sodium dodecyl sulfate (SDS). SDS weakens non-covalent hydrophobic interactions within and among proteins, increasing the exposure of buried thiols without altering disulfide bonds. MS/MS analysis of proteolytic fragments from the isolated AuNP-protein covalent complexes allowed identification of the bound proteins. Proteomics data suggests that AuNPs also associate with gluten proteins lacking free thiols in their native structure, which are bound to AuNPs by forming disulfide bonds with other gluten proteins containing accessible thiols, via thiol-disulfide exchange reactions. This implies that thiol-disulfide reshuffling among gluten proteins occurs already in the grain, enabling proteins without free thiols to become part of AuNP-bound assemblies and revealing specific protein species involved in these early interactions. These observations highlight the role of thiol–disulfide exchange within the grain matrix, elucidating how such molecular rearrangements influence the topology and strength of protein networks in food and in related biopolymeric systems. Results of this exploratory study are discussed for their molecular relevance and for the potential use of size-based analytical and structural approaches in other biological contexts.

## 1. Introduction

It has long been known that gliadins and glutenins in flour from common wheat and in semolina from durum wheat form a cohesive gluten network when mixed in the presence of an appropriate amount of water [1]. The addition of water acts as the primary trigger for molecular reorganization within the wheat protein matrix, promoting novel intermolecular interactions and enabling thiol–disulfide exchange reactions that contribute to the formation and dynamic remodeling of the cohesive gluten network [2]. Upon hydration, both gliadins and glutenins swell from an almost dry and structurally rigid state to a hydrated and flexible form. This enhanced flexibility enables the exposure of key functional groups located on amino acid sidechains (particularly hydrophobic residues and cysteine thiols) that are otherwise buried in the dry matrix. Once exposed, these functional groups mediate the intermolecular interactions responsible for gluten network formation. Non-covalent hydrophobic interactions promote the initial association and spatial organization of gliadin and glutenin subunits, forming a cohesive matrix, whereas covalent disulfide bonds between cysteine residues establish cross-links that stabilize the three-dimensional network. The balance between non-covalent hydrophobic interactions and covalent disulfide bonds determines the viscoelastic properties of the gluten structure. Mechanical shear applied during mixing and subsequent processing steps further reorganizes the protein structure, leading to the development and stabilization of the protein network that characterizes staple foods such as bread, baked goods, noodles, and pasta [3].

The molecular bases of the different behavior of flour and semolina, as well as the molecular determinants of the physical features of the gluten network, have been investigated very thoroughly [4,5,6,7,8,9]. Several reports have tried to address the role of specific proteins or protein fractions in making a given starting material best suited for being processed into a given product. Quite obviously, the easy-to-assess overall protein content is often taken as the discriminating factor, but determining the optimal processing conditions requires a more detailed understanding of the role played by individual components and of their interactions.

Molecular studies in this area primarily investigate the selective disruption of the two key interactions that contribute to the gluten network’s formation, i.e., non-covalent hydrophobic interactions and covalent inter-protein disulfide bonds. Hydrophobic interactions are susceptible to chaotropes and detergents, while disulfide bonds are sensitive to appropriate reductants, typically after protein unfolding [10]. These two types of interactions are rearranged in various steps of the apparently simple path that turns flour into dough or semolina into dry pasta. However, only a few studies have addressed the structural rearrangements relevant to wheat processing steps without resorting to separation and/or extraction methods, which invariably destroy whatever structure the involved proteins may have originally or may acquire upon processing [11,12]. For instance, solid-state fluorescence (also known as front-face fluorescence) has been used to assess changes in protein surface hydrophobicity at various processing stages without any separation step [13]. This method allows for exploring protein structural changes throughout the entire process, from protein deposition in the seed [14] to the solvation of flour/semolina, and through all the steps leading to dried (and then cooked) spaghetti [13].

Yet another non-separative approach to these issues takes into account the distribution of cysteine thiols and disulfide bonds among the various classes and types of gluten-forming proteins [15,16,17], summarized in Table 1. Process-induced changes in thiol accessibility due to intra-molecular and inter-molecular disulfide exchange reactions, to redox events, or to changes in the mutual relationships among proteins involved in network formation have been studied either as a way of discriminating among various materials or to elucidate the role of specific proteins (or protein classes) in individual processing steps or in the finished products [11]. Noteworthily, approaches based on “thiol accessibility” measurements were also able to provide some information about protein structural features as related to interactions with other components of the system, such as starch and non-starch polysaccharides, as well as with non-wheat proteins [18,19].

**Figure 1:**
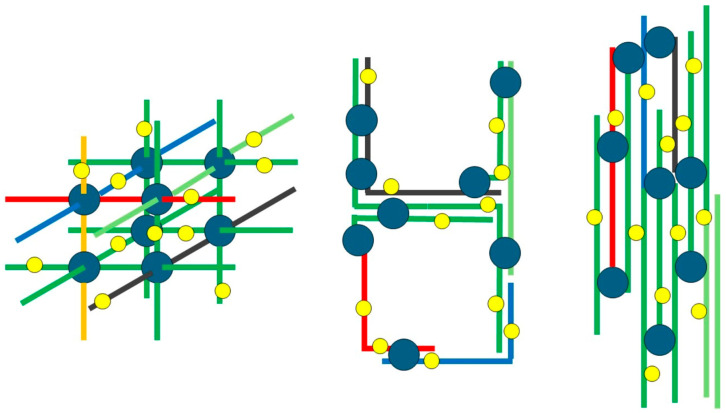
A highly schematic representation of different geometries of protein networks involving the same type and amount of individual polypeptides (in different colors), but resulting in different accessibility of free protein thiols to reagents of different sizes. Blue circles represent interprotein network “knots” (covalent disulfide bonds and/or non-covalent hydrophobic interactions). Yellow dots represent free thiols. For the sake of comparison, all the structures involve the same number of free thiols and of interprotein interactions.

Studies on the location and accessibility of protein thiols in wheat-based systems most frequently rely on the use of small thiol reagents to measure thiol accessibility in the presence or in the absence of dissociating and/or unfolding agents (such as chaotropes or detergents). Thiol-reactive fluorophores have also been used for covalent labeling of exposed thiols in various starting materials and/or at various processing stages. In this latter case, image analysis of bi-dimensional electrophoretic patterns may be used to identify the labeled species [20]. Both approaches use relatively small thiol reagents that are not expected to discriminate among thiols in networks that may differ in their geometry when it comes to mutual spatial relationships among different protein chains, such as those coarsely schematized in Figure 1.

A potential approach to circumventing these limitations and addressing geometrical challenges in protein networks is to use thiol-reactive species that are considerably larger than single-molecule reagents. Thiol-reactive species larger than the Ellman’s reagent (5,5′-dithiobis-2-nitrobenzoic acid, DTNB, MW 396.35 g/mol) or fluorescein derivatives (5-iodoacetamido fluorescein, IAF, MW 515.25 g/mol) should be able or unable to penetrate the gluten network as a function of its compactness (be that dictated by the number of proteins per unit volume and/or by the abundance of interprotein bonds) along with the spatial relationships among individual components [21].

**Figure 2:**
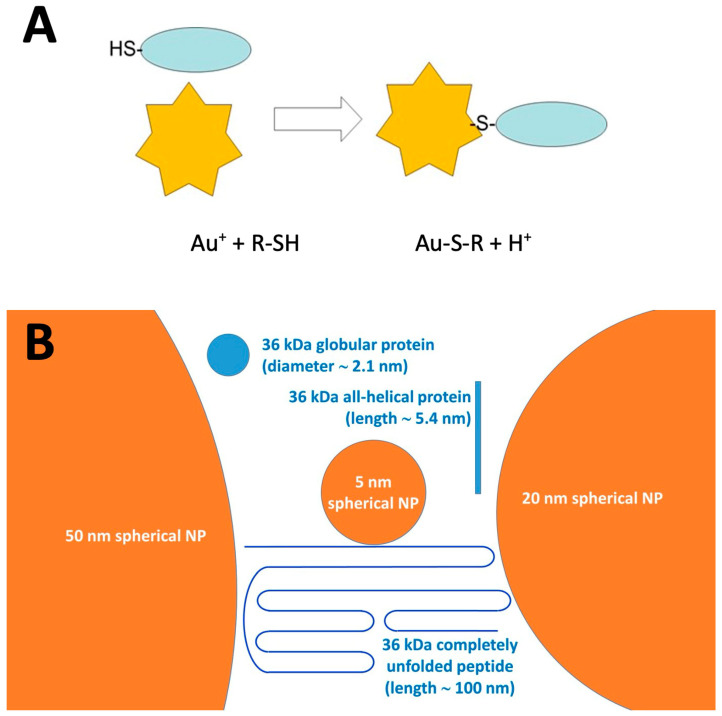
Panel (**A**) chemical reactivity of AuNPs (yellow stars) towards protein-free thiols (blue oval). The gold ions (Au^+^) on the AuNP surface react with the thiol group of a protein cysteine residue (R-SH); this covalent bonding reaction leads to the formation of a highly stable Au-S bond. Panel (**B**) physical traits of NPs (orange circles) and of a hypothetical “average” protein in different conformational states (represented as a blue circle or a blue line).

Thiol-reactive gold nanoparticles (AuNPs) [22,23] offer a powerful and versatile approach to probe the three-dimensional organization of the protein network by targeting accessible thiol groups. AuNPs are commercially available as suspensions of spherical particles with diameters ranging from less than 2 nm to 400 nm, with citrate ions being most frequently used as stabilizers. As illustrated in Figure 2A, residual Au^+^ ions on the AuNP surface rapidly and irreversibly bind to thiols [23]. The resulting Au-S bond is completely insensitive to redox events and to competition from excess thiols that may be added to the system after binding. As depicted in Figure 2B, NPs are relatively close in size to a generic protein with a molecular mass in the 20–100 kDa range and are orders of magnitude bulkier than “single molecule” thiol reagents such as IAF or DTNB. Wheat scientists should also note that the average wheat starch granule has a diameter in the range of 1000 nm (1 micron), and should appear as an enormous—and apparently flat—surface on the scale used in Figure 2B. Other key advantages of AuNPs include their excellent colloidal stability and ease of recovery by centrifugation, enabling reproducible and robust measurements [24].

In this study, the reactivity of AuNPs towards thiols in semolina proteins was exploited to assess whether AuNPs can be used to evaluate the accessibility of the free thiols in native gluten networks. The rationale was that a comparison of results obtained with reagents of different size (from small molecules such as IAF to NPs of increasing size) should be able to provide information on: (1) the geometrical features of the network in the starting material, that affects the accessibility of individual thiols to the reagent; (2) the occurrence of covalent inter-protein interactions (i.e., disulfide bonds), that can be disrupted by disulfide reductants—at difference with the Au-S-protein bonds; (3) the nature of the proteins that were most significant to or most involved in any of the aforementioned events. Altogether, thiol-reactive AuNPs constitute a uniquely stable, size-tunable, and analytically traceable probe that enables the molecular-level assessment of thiol accessibility and protein–protein interactions across the gluten networks. This approach advances the structural understanding of gluten organization beyond what can be achieved with traditional small-molecule reagents or non-specific labeling techniques.

## 2. Materials and Methods

### 2.1. Materials

Durum wheat semolina was obtained from local sources, milled in a laboratory mill (IKA A11 basic Analytical mill, IKA, Staufen, Germany), and sieved through a 0.2 mm sieve. The semolina had a protein content of 13.2% (db), and a gluten quality of 88, as evaluated by the Gluten Index direct method (ICC 158, 1995) [25] carried out by using a Perten Glutomatic System (Perkin-Elmer, Shelton, CT, USA).

Spherically shaped, citrate-stabilized AuNPs (20 nm nominal size) were gently provided by Dr. Laura Polito (CNR-SCITEC, Milan, Italy), who synthesized and characterized them according to published procedures [23,26]. The AuNPs concentration was assessed by using an extinction coefficient of 9.2 × 10^8^ M^−1^ cm^−1^ at 520 nm [27,28]. The stock AuNP suspension used in this study was calculated to contain about 1.3 × 10^11^ individual nanoparticles/mL.

All chemicals were of analytical or higher grade and, unless stated otherwise, were obtained from Sigma (St Louis, MO, USA).

### 2.2. Thiol Labeling

**Figure 3:**
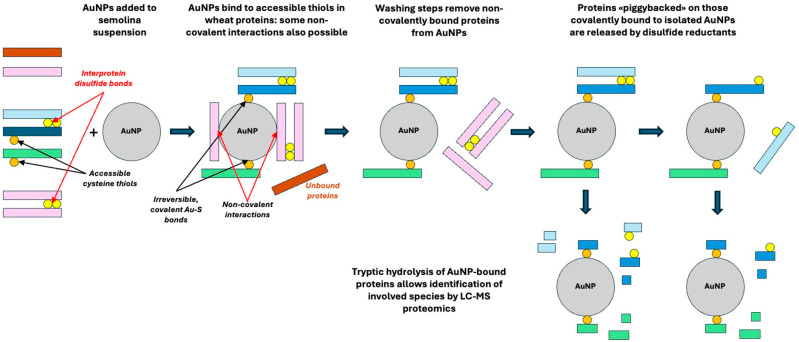
A schematic view of the workflow used for recovery and identification of proteins covalently bound to AuNPs.

Labeling was adapted from Marengo et al. [21]. Fifty milligrams of semolina were suspended in 1 mL of 0.1 M sodium phosphate buffer, pH 7.2, containing 0.1 M NaCl. Where indicated, the suspension buffer also contained 1% SDS. All steps were carried out at room temperature. As for AuNPs, 0.05 mL of a stock AuNP suspension (corresponding to 6.5 × 10^9^ nanoparticles) was added to the semolina suspension, and the mixture was stirred overnight. The mixture was then centrifuged at low speed (800× *g*, 5 min) to remove the swollen semolina granules. AuNPs in the supernatant were then isolated by high-speed centrifugation (10,000× *g*, 30 min, Centrifuge 5425 R, Eppendorf, Milan, Italy). AuNPs were isolated from reaction mixtures prepared in the absence or presence of the detergent (SDS) to disrupt non-covalent hydrophobic interactions within and among proteins, thereby enhancing the exposure of free protein thiols and improving the thiol labelling efficiency. The isolated NPs were then washed by suspending them in 1 mL of 60% (*v*/*v*) aqueous ethanol in the absence/presence of 10 mM dithiothreitol (DTT), followed by extensive sonication (ten 5 s cycles, at 10 s intervals; Soniprep 150, MSE Supplies, Tucson, AZ, USA), and by centrifugation (10,000× *g*, 30 min; Centrifuge 5425 R, Eppendorf, Milan, Italy). DTT has reportedly no effects on the covalent Au-S-protein bonds, and blocks any residual reactive Au^+^ ion on the Au-NP surface. The washing/sonication procedure was repeated three times, and the washed AuNP pellet was frozen and stored at −24 °C prior to MS analysis. The protein profile in supernatants from individual washing/sonication steps was characterized by SDS-PAGE. In separate control experiments, AuNPs were also added to the supernatant from low-speed centrifugation of semolina suspensions prepared in the presence and in the absence of 1% SDS and left overnight at room temperature. The rationale for the various isolation steps is schematized in Figure 3.

### 2.3. SDS-PAGE

Proteins released from AuNP suspensions in the various washing steps described above and schematized in Figure 3 were analyzed by SDS-PAGE after mixing an aliquot of the supernatant from each centrifugation step with an equal volume of denaturing buffer (0.125 mol/L Tris–HCl, pH 6.8; 50% glycerol (*w*/*v*); 1.7% SDS (*w*/*v*); 0.01% Bromophenol Blue (*w*/*v*)), followed by treatment at 100 °C for 5 min. Electrophoretic runs were performed at pH 8.3 (0.025 mol/L Tris–HCl, 0.192 mol/L glycine, 0.1% (*w*/*v*) SDS), on a 12.5% polyacrylamide gel in a Miniprotean II cell (Bio-Rad Laboratories, Hercules, CA, USA), at a constant 16 mA. Gels were stained with Coomassie Brilliant Blue [29].

### 2.4. Trypsin Hydrolysis of AuNP-Bound Proteins

Dried AuNPs from the washing steps reported above were suspended in 0.2 mL of 100 mM ammonium bicarbonate (AmBic), 1 mM CaCl_2_ (pH 8.0). AuNP-bound proteins were digested in duplicate (16 h, 37 °C) with proteomics-grade porcine trypsin (Sigma T6567, theoretical enzyme/substrate ratio 1:100 (*w*/*w*)). Formic acid (FA) was added to a final concentration of 0.1% (*v*/*v*) to stop the reaction. The peptide mixtures in the supernatant of high-speed centrifugation (7900× *g*, 5 min; Microcentrifuge Multispin 12, Steroglass, Perugia, Italy) were desalted on C18 Sep-Pak cartridges (Waters, Milford, MA, USA) following the manufacturer’s instruction, concentrated with gaseous nitrogen, and resuspended in 0.1% (*v*/*v*) aqueous formic acid (FA) prior to analysis by high resolution LC-MS/MS.

### 2.5. High-Resolution LC-MS/MS Analysis

Mass spectrometry analyses were performed by using an EXPLORIS 240 Orbitrap mass spectrometer coupled to a Vanquish-Neo UHPLC system (Thermo Scientific, San Jose, CA, USA). After loading, the peptide mixture was first concentrated and desalted on the pre-column (PepMap™ Neo Trap cartridge). Peptides were separated on a C18 Reverse Phase capillary column (Double nanoViper™ PepMap™ Neo 2 µm C18 75 µm × 150 mm) at a flow rate of 250 nL/min with a gradient from 5% to 95% of solvent B (0.2% FA in 95% acetonitrile (ACN)) over 77 min. Solvent A was 0.2% FA and 2% ACN in ultrapure water.

### 2.6. LC-MS/MS Data Acquisition

The MS/MS acquisition method was set up in a data-dependent acquisition mode with a full scan in the 300 to 1800 *m*/*z* range. Up to 10 of the most intense ions in MS1 were selected for fragmentation in MS/MS mode. A resolving power of 70,000 full-width at half maximum (FWHM), an automatic gain control (AGC) target of 1 × 10^6^ ions, and a maximum ion injection time (IT) of 120 ms were set to generate precursor spectra. Up to 10 of the most intense MS1 ions per cycle were selected for fragmentation in MS/MS mode. MS/MS fragmentation spectra were obtained at a resolving power of 17,500 FWHM. To prevent repeated fragmentation of the most abundant ions, a dynamic exclusion of 10 s was applied. Singly charged ions or ions with charge states > 4 were excluded from fragmentation.

### 2.7. Bioinformatic Analysis

MS spectra were processed by using the Xcalibur Software version 3.1, and then analysed using Protein Prospector version 6.4.9 (Thermo Scientific, San Jose, CA, USA). The analysis of the mass spectra was performed using *Triticum* as a background database (369,806 entries) (UniProtKB. 2020.09.02.random.concat). Search settings included: 8 ppm precursor and 0.01 Da fragment mass tolerances, trypsin digestion, variable methionine oxidation, glutamine/asparagine deamidation, N-terminal pyroglutamate formation, and presence of disulfides. A 1% peptide FDR was applied, with minimum scores of 22 for proteins and 15 for peptides. Entries annotated as “fragment” were retained to improve coverage of the AuNP-associated proteome. Analyses were performed in biological duplicates, and gluten-related datasets were merged after removing duplicates.

### 2.8. Statistical Analysis

The experimental design is a full factorial with 2 variables (binding in phosphate buffer or phosphate buffer/SDS 1%) at 2 independent levels (washing in ethanol 60% or ethanol 60%/DTT 10 mM) for a total of 2^2^ = 4 trials [30].

The statistical analysis was performed using the SPSS software package, version 30.0 (IBM, Armonk, NY, USA). Analysis of variance (ANOVA) to verify mean differences between the number of proteins identified was applied, followed by Tukey’s Honest Significant Difference (HSD) with a statistical significance set to 0.05.

## 3. Results

The study presented here was carried out on a thoroughly characterized semolina in the form of an aqueous suspension of particles of uniform size in water-based systems, thus avoiding any mechanical stress on the protein network. Suspensions were prepared in buffer systems that allow different degrees of swelling/relaxation of the protein structure and of the network they may form in semolina, namely: (1) buffered saline alone, leading to hydration of polar regions and weakening of electrostatic bonds in proteins; (2) buffered saline containing 1% SDS, leading—at room temperature—to weakened hydrophobic interactions.

From a methodological standpoint, the approach reported here is conceptually simple and takes advantage of the high density of AuNPs as well as of the relative stability of their suspensions. As reported in Figure 3, suspensions of flour are allowed to interact with AuNPs at room temperature. Non-reactive insoluble wheat proteins are then removed in a first low-speed centrifugation step, which leaves AuNPs and soluble proteins in suspension. AuNPs can then be recovered by high-speed centrifugation and washed to remove non-covalently bound proteins. Finally, proteolytic treatment of the AuNPs is used to release peptides from the species covalently bound to the AuNPs surface, and MS may be used to assess the nature of the proteins that remained grafted on the AuNPs surface through Au-S bonds. If required, a treatment with DTT (or 2-ME) may be included in the sample workflow prior to trypsinolysis and MS identification. Treatment with disulfide-reducing agents does not remove AuNP-bound proteins, but allows the release of any protein that may be “piggybacked” by intermolecular disulfides on the ones covalently bound to AuNPs.

The size of AuNPs to be used in this study was chosen on the basis of the relative size of the particles and of the proteins expected to be captured, taking into account also the possibility that some of the target proteins may be present in a partially unfolded form (see Figure 2). AuNPs in this size range also offer some practical advantages, such as the long-term stability of their suspensions and the ease of separating them from other components of the reaction mixture through simple centrifugation steps. Larger AuNPs could end up in the swollen semolina granules precipitate in the earliest recovery steps regardless of the conditions used for interaction, whereas isolation of AuNPs smaller than 10 nm in diameter reportedly requires lengthy and laborious ultracentrifugation steps [23,31].

In order to distinguish between thiols from soluble proteins (durum wheat albumins and globulins) and from proteins that become soluble only in the presence of detergents, AuNPs were also added to the supernatant obtained by low-speed centrifugation of semolina suspensions in buffered saline, with or without 1% SDS. These experiments served as “control runs”, where AuNPs were expected to react with any exposed cysteine thiols in water-soluble proteins, regardless of their folding state or non-covalent interprotein interactions.

High-resolution LC-MS/MS analysis of semolina proteins selectively captured by thiol-reactive AuNPs provides a clear picture of which proteins can bind to the particles. A comprehensive listing of all the proteins and peptides detected under the various experimental conditions used in this study is presented in Table S2 in the Supplementary Materials, and a list of the disulfide bonds identified in the various samples is provided in Table S3. The protocol outlined in Figure 3 was effective in limiting contamination of the recovered AuNPs by adventitiously bound proteins. SDS-PAGE tracings [21] indicated that the proteins removed from the AuNPs by washing in the absence/presence of detergents and chaotropes (i.e., urea 6M) did not contain appreciable amounts of species relevant to gluten formation, as already reported in Marengo et al. [21].

Table 2 lists the gluten proteins recovered from AuNPs incubated with semolina suspensions prepared either in plain buffered saline or in the presence of a detergent (1% SDS). Only a restricted subset of gluten proteins is found associated with nanoparticles (13 entries) in the absence of the detergent, whereas the addition of 1% SDS markedly increases both the number and the diversity of AuNP-associated gluten species (28 entries). This pattern is consistent with an SDS-driven loosening of hydrophobic contacts in semolina, which increases the accessibility of reactive thiol groups to the bulky AuNPs.

Species captured in both presence and absence of 1% SDS included gamma-gliadin (UniProtKB ID: F2X0K8) and a prolamin (UniProtKB ID: Q0GK30) sharing 97.2% identity with *T. aestivum* alpha/beta gliadin (UniProtKB ID: P02863). Also present in bound form in both conditions were low molecular weight (LMW) glutenin species (UniProtKB ID: A2IBV5) and two high molecular weight (HMW) glutenin subunits (UniProtKB IDs: A0A2L1K3K6 and Q41553). This suggests these proteins possess a relatively accessible/loose native conformation that facilitates rapid reaction with AuNPs regardless of the presence of the detergent.

The data in Table 3 provide additional peptide-level evidence for many of the AuNP-bound glutenin species retaining intact intramolecular disulfide bonds, as many identified peptide pairs unambiguously contain disulfide-linked cysteines (see also Table S3 in the Supplementary Materials). Thus, covalent attachment to AuNPs through surface-exposed protein thiols can coexist with preservation of intramolecular disulfide topology in many glutenin molecules, implying that nanoparticle binding does not require (or induce) disruption of native disulfide bonds.

Taken together, the data in Table 2 and Table 3 support a model in which a subset of glutenin molecules present free or partially exposed thiols that become available for covalent binding to the nanoparticle surface once local hydrophobic packing is loosened, leaving intramolecular disulfide bonds intact [32,33]. Limited local rearrangements of hydrophobic contacts can be sufficient to expose a reactive cysteine for Au-S binding without breaking the intramolecular disulfide framework [34]. The peptide-level assignments in Table 3 provide molecular evidence that AuNPs can bind to proteins through accessible thiols while preserving native intra-chain disulfide frameworks, indicating that binding occurs without the prior reduction of S-S bridges.

Table 4 provides additional information not only on gluten-related proteins directly attached to the nanoparticle surface by a covalent Au-S bond, but also on those “piggybacked” on AuNP-bound proteins (i.e., associated indirectly with AuNPs through intermolecular disulfide linkages to directly bound proteins). This distinction was introduced to specifically capture and differentiate proteins directly bound to AuNPs from those associated indirectly via intermolecular disulfide linkages, allowing us to better interpret the network of AuNP-protein interactions. This information comes from a comparison between proteins remaining associated with AuNPs after an ethanol wash (Table 4, left column), with those still present after the ethanol wash was followed by a disulfide-breaking treatment with 10 mM DTT (Table 4, right column). The comparison makes it evident that a cohort of proteins—including several gliadins—is lost or strongly depleted after the DTT treatment, indicating that they are “piggybacked” species, bound to AuNPs through intermolecular disulfide interactions with proteins that form direct Au-S bonds. Notably, a set of glutenins (9 entries) remains associated with AuNPs even after treatment with DTT, indicating either a direct Au-S attachment or a binding tight enough to resist high ethanol concentrations.

LMW gliadins—with two free thiols in their structure (see Table 1)—represent the most likely candidates for direct binding to AuNPs. Noteworthily, also 1Ax1 and 1Bx20 HMW-glutenin subunits (UniProtKB IDs: Q03872 and Q8RVX0), and two y-type HMW glutenin subunits (UniProtKB IDs: Q03871, Q0Q5D8 and A3RF26) were still present as AuNP-bound species after the ethanol-DTT washing steps. Reportedly, y-type HMW glutenins have an odd number of Cys residues (see Table 1), making them reliable candidates for direct binding to AuNP. Other HMW types have an even number of Cys residues, so that they can only be piggybacked on either y-type or LMW glutenins (as gliadins do) or have been previously crosslinked to other gluten proteins through thiol-disulfide exchange reactions.

**Figure 4:**
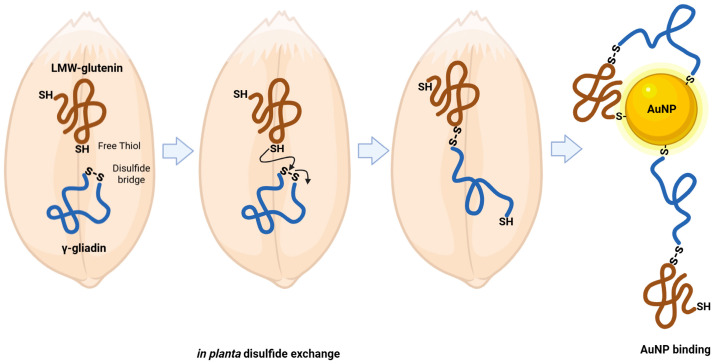
A schematic view of thiol–disulfide exchange reactions involving disulfide bonds in gamma-gliadins and free thiols in LMW-glutenins, as relevant to covalent binding to AuNPs.

In this frame, the persistence of gamma-gliadin (F2X0K8) after the EtOH/DTT sequential washing steps deserves particular attention. This protein, which lacks free cysteine residues (see Table 1), was detected in a form directly bound to AuNPs. This observation is consistent with the idea that gamma-gliadin may already have undergone disulfide exchange reactions in the starting semolina, enabling its direct interaction with AuNPs. Notably, in our experimental conditions, no mechanical stress was applied—apart from overnight stirring of a dilute semolina suspension at room temperature. Therefore, the presence of intermolecular disulfide-linked complexes in the starting material suggests that disulfide exchange and crosslinking may occur “in planta” or during early grain/semolina handling, such as milling. A reasonable working hypothesis implies that gliadins in semolina can undergo disulfide exchange with specific glutenin species, notably LMW or y-type HMW glutenins, that possess free thiols suitable for thiol-disulfide reactions that make proteins with no free thiols capable of covalent binding to AuNPs, as schematized in Figure 4.

Previous literature evidence indicates that gamma-gliadins, although typically characterized by intrachain disulfide bonds, can also form intermolecular linkages with glutenins [35]. The resulting oligomeric fraction, called high molecular weight gliadins (HGL), represents a third gluten protein fraction besides monomeric gliadins and polymeric glutenins, accounting for about 10–15% of total gluten proteins [36]. Building on this evidence, an additional working hypothesis proposes that specific mutations in gamma-gliadins may alter their cysteine content, thereby facilitating their integration into the glutenin polymeric network. Supporting this idea, genes encoding gamma-gliadins with a non-canonical number of cysteines have been identified. In particular, Ferrante et al. [37] reported that a gamma-gliadin gene from the durum wheat cultivar Lira biotype 45 contained nine cysteines instead of the usual eight, is expressed “in planta”, and contributes to glutenin polymers. This was demonstrated through heterologous expression in *E. coli*, followed by 2-DE, RP-HPLC, and MS/MS analyses, which confirmed the presence of the additional cysteine in the N-terminal region. It has been hypothesized that the extra cysteine, located at position 26, does not disrupt the typical intramolecular disulfide bond pattern but instead promotes intermolecular bonding, enabling incorporation of the mutant gliadin into gluten polymers as potential gluten chain terminators [38].

**Figure 5:**
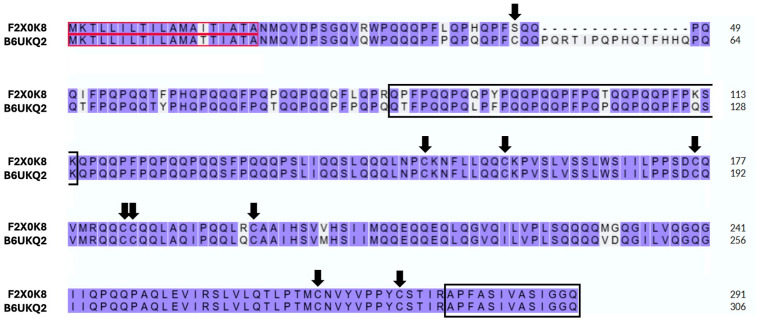
Sequence alignment of gamma-gliadins (UniProtKB IDs: F2X0K8 and B6UKQ2). Arrows indicate cysteine residues. Black outlined boxes highlight the alignment between the peptide sequences identified and the odd-cysteine–containing gamma-gliadin. Red outlined boxes indicate the signal peptides. The alignment was performed using https://www.uniprot.org/align (accessed on 24 November 2023).

In the case of the gamma-gliadin found in this study (UniProtKB ID: F2X0K8, Table 3), we unequivocally identified two peptides: a short one (APFASIVASIGGQ) and a long one (QPFPQQPQQPYPQQPQQPFPQTQQPQQPFPKSK). While the long peptide is unique to this accession, the short one is shared by several other gamma-gliadins, including two entries (UniProtKB IDs: B6UKM7 and B6UKQ2) that possess an odd cysteine at position 26 in the N-terminal region. Since F2X0K8 and B6UKQ2 display a high sequence identity (93.13%) (Figure 5), the hypothetical presence of a modified isoform of F2X0K8 in our samples cannot be ruled out. Consistently, N-terminal sequences of gamma-gliadins have been detected in glutenin preparations, and Lutz et al. [39] provided MS-based evidence that this region of some gamma-gliadins can establish interchain disulfide bonds with LMW-GS and with other gluten proteins. Nonetheless—in our case—this latter interpretation remains somewhat hypothetical, as the identification of the N-terminal gamma-gliadin peptide carrying the additional cysteine cannot be unequivocally confirmed due to its possible covalent binding to AuNPs.

Discussion of the proteomics data in this study has been limited so far to gluten proteins, in view of their practical interest and of the difficulties associated with molecular-level studies on gluten networks. The various panels of Figure S1 (in the Supplementary Materials) provide some additional proteomics-based information on all the proteins released upon addition of a disulfide reductant to AuNP-bound proteins (including albumins and globulins, see Table S2). The data in Figure S1, with reference to the samples listed in Table S1, clearly show that the inclusion of a protein-swelling agent (i.e., 1% SDS) results in a significant increase (*p* < 0.05) in the number of insoluble proteins captured on the AuNP surface, confirming—on an expanded scale—what observed for gluten-related proteins as discussed above. As expected, disulfide reductants like DTT do not affect the Au-S covalent bond itself, but they can disrupt intermolecular disulfide linkages between proteins directly bound to AuNPs and those associated indirectly through such crosslinks.

Given the exploratory nature of our study and the very low amount of AuNPs used here, we did not assess in this study the amount of AuNPs remaining in the insoluble materials after low-speed centrifugation, or the nature of the insoluble proteins the ‘insolubilized’ AuNPs may have stuck to. This could be of some interest in the specific network studied here, since non-soluble materials may include proteins that were not detachable from the remainder of the network, and these proteins may be expected to be a large part of the water-insoluble proteins in semolina and flour.

Conversely, characterization of the proteins remaining insoluble, and identification of proteins bound to AuNPs in the low-speed centrifugation insoluble fraction will require some ingenuity, in consideration of the small amount of AuNPs present in the system with respect to proteins. Although outside the scope of this report, these approaches may take advantage of the use of disulfide reducing agents in the presence of either detergents or chaotropes to solubilize the remaining proteins. In this frame, the use of thiol-based disulfide reductants offers some additional experimental convenience, as these reductants block residual reactive Au^+^ ions on the AuNPs surface and prevent further reactivity of the AuNPs surface towards protein thiols.

## 4. Discussion and Conclusions

The accessibility of thiols in gluten proteins to reactive Au ions on the surface of AuNPs was studied on semolina suspensions in the absence of thermal or mechanical treatments. AuNPs’ access to thiols in gluten proteins was greatly improved in the presence of 1% SDS. Even at room temperature, the ability of the anionic detergent to loosen hydrophobic interactions in semolina resulted in facilitated exposure of additional protein thiols in proteins involved in gluten formation without the need for either mechanical or temperature-induced protein unfolding [40,41].

The use of MS/MS analysis—after removal of adventitious material—allowed the identification of most of the proteins covalently bound to AuNPs, as well as of those “piggybacked” to covalently bound ones through disulfide bonds. Some of the proteins reacting with AuNPs are species reportedly assumed to have all their thiols involved in intramolecular disulfides. Given the absence of any physical denaturation step in the handling of these samples, it seems evident that inter-molecular disulfide exchange events have already occurred prior to milling *Triticum durum* grains into semolina. Both the timing of intermolecular disulfide formation and the nature of the proteins involved at various protein deposition steps are currently under investigation by using suitable durum wheat lines at various times of seed development. 

As for the relevance of the possible use of thiol accessibility in distinguishing protein networks with different geometries (see Figure 1), it seems evident that AuNPs cannot penetrate the protein network in finely milled semolina in the absence of a treatment with detergents. This is quite different from the thiol accessibility data reported for the same proteins when using thiol orders of magnitude smaller than the AuNPs used here, such as the colorimetric reagent DTNB [11] or the fluorescent label 5-iodoacetamido fluorescein (IAF) [21,29]. In those studies, addition of IAF to semolina suspensions resulted in covalent labeling of durum wheat proteins (as made evident by fluorescent protein spots in 2D electrophoresis) regardless of the addition of 6M urea, indicating that breakdown of hydrophobic interactions in semolina was not required for granting access or the relatively small IAF reagent to the innermost part of whatever network was present in the original semolina. The same studies also indicated that both gliadins and glutenins may be labeled by IAF—as observed here for reactivity towards AuNPs—providing additional evidence for the “in planta” or “in grain” occurrence of disulfide exchange reactions relevant to important properties of the systems.

The above remarks underscore the possibility of using differently sized thiol reagents to address the geometry of water-insoluble protein aggregates relevant to food and health issues. This report proves the feasibility of such an approach, as well as its potential in sorting out the contribution of individual species. This may pave the way to further applications and developments, including some of great interest for the food scientist, as structural alterations of proteins leading to novel interactions among them (or with other food components) are the molecular basis of food-related transformation [42,43]. Geometry-based approaches may also hold promise for breeding and crop science, where they could help to address the challenges of interpreting structural changes during grain development and protein deposition—processes that cannot be fully understood solely in terms of the timing and extent of protein synthesis [44]. Finally, the size-dependent reactivity approaches presented here may contribute to a deeper understanding of process-related modifications in food systems, while also clarifying their molecular determinants and their sensitivity to processing conditions and to the amount and nature of individual ingredients.

## Figures and Tables

**Table 1:** Cysteine thiols and intra-protein disulfide bonds in the as-isolated major families of gluten-forming wheat proteins. Adapted from Bonomi et al. [17].

Protein Family	Individual Proteins	Free Cysteine Thiols			Thiols Involved in Disulfide Bonds		
		N- Terminus	Repeat Region	C- Terminus	N- Terminus	Repeat Region	C- Terminus
Gliadins	Alpha and beta	none	none	none	none	none	6 *
	Gamma	none	none	none	none	none	8 *
	Omega	none	none	none	none	none	none
HMW Glutenins	x-type	none	none	none	3 **	none	1 **
	Dx5	none	none	none	3 **	1 **	1 **
	y-type	none	none	none	5 **	1 **	1 **
LMW Glutenins	s- and m-type	1	none	1	none	none	6 *
	i-type	none	none	2	none	none	6 **

(*) All forming known intra-molecular disulfide bonds; (**) Involved cysteines undetermined because of thiol-disulfide exchange reactions.

**Table 2:** Gluten proteins covalently associated with AuNPs following incubation of semolina suspensions in buffered saline in the absence or presence of 1% SDS. Protein identity was assigned by LC–MS/MS analysis. The table lists UniProt ID, molecular mass and protein family.

Buffer Only				Buffer + 1% SDS			
UniProt ID	Mass	Origin	Protein Name	UniProt ID	Mass	Origin	Protein Name
**Prolamin family**							
				A7LHB5	33,068.8	WHEAT	Alpha-gliadin
				M7ZZV2	23,557.9	TRIUA	Alpha/beta-gliadin MM1
				H6VLP7	32,301.8	WHEAT	Alpha-gliadin
F2X0K8	33,083.4	WHEAT	Gamma-gliadin	F2X0K8	33,083.4	WHEAT	Gamma-gliadin
				A0A2U8JD23	41,892.9	WHEAT	Gamma-gliadin B4
				R9XWC4	37,468.1	WHEAT	Gamma-gliadin
				A7LHB4	33,014.7	WHEAT	Alpha-gliadin
				P04726	33,941.6	WHEAT	Alpha/beta-gliadin clone PW1215
				A0A023WHQ9	32,897.4	WHEAT	Alpha-gliadin
				R9XSV1	32,807.4	WHEAT	Alpha-gliadin
				R9XU90	36,002.7	WHEAT	Alpha-gliadin
				R9XUS6	40,793.6	WHEAT	Gamma-gliadin
Q0GK30	32,803.5	TRITI	Prolamin	Q0GK30	32,803.5	TRITI	Prolamin
				R4JB53	37,803.1	WHEAT	Low-molecular-weight glutenin subunit
				Q42451	84,637.6	WHEAT	Glu-B1-1b HMW glutenin subunit
				A0A1D8V7C6	61,970.4	WHEAT	High molecular weight glutenin subunit
				A0A142ESP2	66,181	TRIDC	High molecular weight glutenin subunit 1Ay protein
P08453	37,122.6	WHEAT	Gamma-gliadin				
Q9M4L9	31,490.7	WHEAT	Alpha-gliadin				
**LMW-Glutenin family**							
A2IBV5	40,435.1	WHEAT	Glutenin subunit	A2IBV5	40,435.1	WHEAT	Glutenin subunit
				B2BZC7	32,721.7	WHEAT	LMW-m glutenin subunit 0154A5-M
				Q9FEQ2	44,567.1	TRITD	Low molecular weight glutenin subunit (Fragment)
				A0A0S2GJR1	39,893.7	WHEAT	Low-molecular-weight glutenin subunit
Q0GQX1	33,978.8	WHEAT	Low molecular weight glutenin subunit (Fragment)				
O49958	39,791.5	TRITD	Low molecular weight glutenin subunit (Fragment)				
**HMW-Glutenin family**							
A0A2L1K3K6	73,890.6	TRITD	High molecular weight glutenin (Fragment)	A0A2L1K3K6	73,890.6	TRITD	High molecular weight glutenin (Fragment)
				A0A2P1E384	66,122.9	TRIDC	High molecular weight glutenin subunit 1Ay
				Q8RVX0	86,038.3	TRITD	High molecular weight glutenin subunit 1Bx20 (Fragment)
				A0A0K0KDM6	85,285.7	WHEAT	High molecular weight glutenin subunit 1Dy3
				A0A142BX75	10,150.8	TRITD	High-molecular-weight glutenin subunit (Fragment)
Q41553	88,476.2	WHEAT	HMW glutenin subunit Ax2	Q41553	88,476.2	WHEAT	HMW glutenin subunit Ax2
				A3RF26	32,042.5	WHEAT	Truncated high molecular weight glutenin subunit 1By9
A0A1D8V7C6	61,970.4	WHEAT	High molecular weight glutenin subunit				
Q03872	89,819.5	WHEAT	High molecular weight glutenin subunit 1Ax1				
Q18MZ6	80,069.8	WHEAT	High-molecular-weight glutenin subunit Bx17				
Q0Q5D8	77,315.6	WHEAT	High-molecular-weight glutenin By8				

**Table 3:** Peptide-level evidence of intramolecular disulfide-bonded cysteines in gluten proteins covalently bound to AuNPs. Identified peptide pairs demonstrate that Au–S attachment can occur without disrupting native intramolecular disulfide topology.

UniProt ID	Peptide 1	Peptide 2	Protein Name
A0A2L1K3K6	C(+Disulfide)RPVAVSQVVR	Q(Gln->pyro-Glu)LQC(+Disulfide)ER	High molecular weight glutenin (Fragment)
A0A2L1K3K6	C(+Disulfide)RPVAVSQVVR	QLQC(+Disulfide)ER	High molecular weight glutenin (Fragment)
A0A2P1E384	C(+Disulfide)RPVALSQVAR	QLQC(+Disulfide)ER	High molecular weight glutenin subunit 1Ay
Q9FEQ2	VFLQQQC(+Disulfide)IPVAMQR	SQMLQQSIC(+Disulfide)HVMQR	Low molecular weight glutenin subunit (Fragment)
A0A0S2GJR1	TLPTMC(+Disulfide)SVNVPVYGTTTIVPFGVGTR	VFLQQQ(Deamidated)C(+Disulfide)SPVAMPQSLAR	Low-molecular-weight glutenin subunit

**Table 4:** Comparison of gluten proteins directly bound to AuNPs versus those associated through intermolecular disulfide linkages (“piggybacked” proteins). Proteins remaining after ethanol wash are listed in the left column; those persisting after additional treatment with 10 mM DTT are shown in the right column.

NP-Bound Gluten Proteins After EtOH Wash				NP-Bound Gluten Proteins After EtOH/DTT Wash			
UniProt ID	Mass	Origin	Protein Name	UniProt ID	Mass	Origin	Protein Name
**Prolamin family**							
A7LHB5	33,068.8	WHEAT	Alpha-gliadin				
M7ZZV2	23,557.9	TRIUA	Alpha/beta-gliadin MM1				
H6VLP7	32,301.8	WHEAT	Alpha-gliadin				
F2X0K8	33,083.4	WHEAT	Gamma-gliadin	F2X0K8	33,083.4	WHEAT	Gamma-gliadin
A0A2U8JD23	41,892.9	WHEAT	Gamma-gliadin B4				
R9XWC4	37,468.1	WHEAT	Gamma-gliadin				
A7LHB4	33,014.7	WHEAT	Alpha-gliadin				
P04726	33,941.6	WHEAT	Alpha/beta-gliadin clone PW1215				
A0A023WHQ9	32,897.4	WHEAT	Alpha-gliadin				
R9XSV1	32,807.4	WHEAT	Alpha-gliadin				
R9XU90	36,002.7	WHEAT	Alpha-gliadin				
R9XUS6	40,793.6	WHEAT	Gamma-gliadin				
Q0GK30	32,803.5	TRITI	Prolamin				
**LMW-Glutenin family**							
				P10385	41,020.4	WHEAT	Glutenin, low molecular weight subunit
				O49958	39,791.5	TRITD	Low molecular weight glutenin subunit (Fragment)
R4JB53	37,803.1	WHEAT	Low-molecular-weight glutenin subunit				
A2IBV5	40,435.1	WHEAT	Glutenin subunit	A2IBV5	40,435.1	WHEAT	Glutenin subunit
B2BZC7	32,721.7	WHEAT	LMW-m glutenin subunit 0154A5-M				
Q9FEQ2	44,567.1	TRITD	Low molecular weight glutenin subunit (Fragment)				
A0A0S2GJR1	39,893.7	WHEAT	Low-molecular-weight glutenin subunit				
**HMW-Glutenin family**							
				Q03872	89,819.5	WHEAT	High molecular weight glutenin subunit 1Ax1
				Q03871	75,701.9	WHEAT	HMW glutenin subunit 1By9
				Q0Q5D8	77,315.6	WHEAT	High-molecular-weight glutenin By8
A0A1D8V7C6	61,970.4	WHEAT	High molecular weight glutenin subunit				
A0A142ESP2	66,181	TRIDC	High molecular weight glutenin subunit 1Ay protein	A0A142ESP2	66,181	TRIDC	High molecular weight glutenin subunit 1Ay protein
Q42451	84,637.6	WHEAT	Glu-B1-1b HMW glutenin subunit				
A0A2L1K3K6	73,890.6	TRITD	High molecular weight glutenin (Fragment)				
A0A2P1E384	66,122.9	TRIDC	High molecular weight glutenin subunit 1Ay				
Q8RVX0	86,038.3	TRITD	High molecular weight glutenin subunit 1Bx20 (Fragment)	Q8RVX0	86,038.3	TRITD	High molecular weight glutenin subunit 1Bx20 (Fragment)
A0A0K0KDM6	85,285.7	WHEAT	High molecular weight glutenin subunit 1Dy3				
A0A142BX75	10,150.8	TRITD	High-molecular-weight glutenin subunit (Fragment)				
Q41553	88,476.2	WHEAT	HMW glutenin subunit Ax2				
A3RF26	32,042.5	WHEAT	Truncated high molecular weight glutenin subunit 1By9	A3RF26	32,042.5	WHEAT	Truncated high molecular weight glutenin subunit 1By9

## Data Availability

The original contributions presented in this study are included in the article/Supplementary Materials. Further inquiries can be directed to the corresponding author.

## Supplementary Materials

The following supporting information can be downloaded at: https://www.mdpi.com/article/10.3390/foods14233985/s1, Table S1: Description of experimental samples used for AuNP-protein binding analysis. Semolina suspensions or extracts were incubated with gold nanoparticles (AuNPs) in cold buffered saline or cold buffered 1% SDS, followed by washing with ethanol (EtOH) or ethanol/dithiothreitol (EtOH/DTT); Table S2: List of proteins and peptides identified across the different experimental conditions used in this study; Table S3: MS identification of disulfide-bonded peptides identified across the different experimental conditions used in this study; Figure S1: Characterization of nanoparticle (NP)-bound proteins under different extraction and washing conditions. (A) Number of proteins and peptides bound to gold nanoparticles (AuNPs) after sequential washing steps (EtOH or EtOH/DTT) in cold buffered saline versus cold buffered 1% SDS, comparing protein extracts and suspensions. (B) Quantification of AuNP-bound proteins identified across samples (A-L) under the two buffer conditions. Bars represent duplicate analyses and their intersection (1, 2,C). (C) Schematic representation of AuNP–protein interactions: initial binding of proteins with accessible thiols, removal of non-thiol proteins after washing, and release of disulfide-bound proteins upon treatment with reducing agents (DTT or TCEP).

## Abbreviations

The following abbreviations are used in this manuscript:

2-ME	2-mercaptoethanol
ACN	Acetonitrile
AGC	Automatic gain control
AmBic	Ammonium bicarbonate
AuNP	Thiol-reactive gold nanoparticles
db	Dry basis
DTNB	Ellman’s reagent, 5,5′-dithiobis-2-nitrobenzoic acid
DTT	DL-Dithiothreitol
FA	Formic acid
FWHM	Full width at half maximum
HGL	High molecular weight gliadins
HMW	High molecular weight
IAF	5-iodoacetamido fluorescein
LC-MS/MS	Liquid chromatography-tandem mass spectrometry
LMW	Low molecular weight
NP	Nanoparticles
SDS-PAGE	Sodium dodecyl sulfate-polyacrylamide gel electrophoresis
TRIDC	*Triticum turgidum* subsp. *dicoccoides*
TRITD	*Triticum turgidum* subsp. *durum*
TRITI	*Triticum dicoccon* var. *timopheevii*
TRIUA	*Triticum Urartu*
UHPLC	Ultra-high-performance liquid chromatography
WHEAT	*Triticum aestivum*

## References

[1] Shewry P. (2019). What is gluten—Why is it special?. Front. Nutr..

[2] Lancelot E., Fontaine J., Grua-Priol J., Assaf A., Thouand G., Le-Bail A. (2021). Study of structural changes of gluten proteins during bread dough mixing by Raman spectroscopy. Food Chem..

[3] Wieser H., Koehler P., Scherf K.A. (2023). Chemistry of wheat gluten proteins: Qualitative composition. Cereal Chem..

[4] Belton P.S. (1999). On the elasticity of wheat gluten. J. Cereal Sci..

[5] Singh H., MacRitchie F. (2001). Application of polymer science to properties of gluten. J. Cereal Sci..

[6] Shewry P.R., Halford N.G., Belton P.S., Tatham A.S. (2002). The structure and properties of gluten: An elastic protein from wheat grain. Phil. Trans. R. Soc. B Biol. Sci..

[7] Veraverbeke W.S., Delcour J.A. (2002). Wheat protein composition and properties of wheat glutenin in relation to breadmaking functionality. Crit. Rev. Food Sci. Nutr..

[8] Goesaert H., Brijs K., Veraverbeke W.S., Courtin C.M., Gebruers K., Delcour J.A. (2005). Wheat flour constituents: How they impact bread quality, and how to impact their functionality. Trends Food Sci. Technol..

[9] Gobaa S., Bancel E., Branlard G., Kleijer G., Stamp P. (2008). Proteomic analysis of wheat recombinant inbred lines: Variations in prolamin and dough rheology. J. Cereal Sci..

[10] Shewry P.R., Belton P.S. (2024). What do we really understand about wheat gluten structure and functionality?. J. Cereal Sci..

[11] Bonomi F., D’Egidio M.G., Iametti S., Marengo M., Marti A., Pagani M.A., Ragg E.M. (2012). Structure-quality relationship in commercial pasta: A molecular glimpse. Food Chem..

[12] Kłosok K., Welc R., Fornal E., Nawrocka A. (2021). Effects of Physical and Chemical Factors on the Structure of Gluten, Gliadins and Glutenins as Studied with Spectroscopic Methods. Molecules.

[13] Bonomi F., Mora G., Pagani M.A., Iametti S. (2004). Probing structural features of water-insoluble proteins by front-face fluorescence. Anal. Biochem..

[14] Quayson E.T., Marti A., Morris C.F., Marengo M., Bonomi F., Seetharaman K., Iametti S. (2018). Structural consequences of the interaction of puroindolines with gluten proteins. Food Chem..

[15] Scherf K., Shewry P.R., Koksel H., Taylor J.R.N. (2023). Gluten Proteins. ICC Handbook of 21st Century Cereal Science and Technology.

[16] Zang P., Gao Y., Chen P., Lv C., Zhao G. (2022). Recent Advances in the Study of Wheat Protein and Other Food Components Affecting the Gluten Network and the Properties of Noodles. Foods.

[17] Bonomi F., Ferranti P., Mamone G. (2014). Wheat Flour: Chemistry and Biochemistry. Bakery Products Science and Technology: Second Edition.

[18] Caramanico R., Barbiroli A., Marengo M., Fessas D., Bonomi F., Lucisano M., Pagani M.A., Iametti S., Marti A. (2017). Interplay between starch and proteins in waxy wheat. J. Cereal Sci..

[19] Emide D., Magni C., Saitta F., Cardone G., Botticella E., Fessas D., Iametti S., Lafiandra D., Sestili F., Marti A. (2023). Molecular insights into the role of amylose/amylopectin ratio on gluten protein organization. Food Chem..

[20] Magni C., Emide D., Iametti S., Barbiroli A., Ferranti P. (2024). A Thiolomic Approach to Map Free Thiol Distribution in Food Proteins. Proteomics Applied to Foods. Methods and Protocols in Food Science.

[21] Marengo M., Mamone G., Ferranti P., Polito L., Iametti S., Bonomi F. (2019). Topological features of the intermolecular contacts in gluten-forming proteins: Exploring a novel methodological approach based on gold nanoparticles. Food Res. Int..

[22] Boisselier E., Astruc D. (2009). Gold nanoparticles in nanomedicine: Preparations, imaging, diagnostics, therapies and toxicity. Chem. Soc. Rev..

[23] Zhao P., Li N., Astruc D. (2013). State of the art in gold nanoparticle synthesis. Coord. Chem. Rev..

[24] Balasubramanian S.K., Yang L., Yung L.Y.L., Ong C.N., Ong W.Y., Yu L.E. (2010). Characterization, Purification, and Stability of Gold Nanoparticles. Biomaterials.

[25] International Association for Cereal Science and Technology (ICC) (1995). Method No. 158: Gluten Index Method for Assessing Gluten Strength in Durum Wheat (Triticum durum).

[26] Compostella F., Pitirollo O., Silvestri A., Polito L. (2017). Glyco-gold nanoparticles: Synthesis and applications. Beilstein J. Org. Chem..

[27] Haiss W., Thanh N.T.K., Aveyard J., Fernig D.G. (2007). Determination of Size and Concentration of Gold Nanoparticles from UV-Vis Spectra. Anal. Chem..

[28] Liu X., Atwater M., Wang J., Huo Q. (2007). Extinction Coefficient of Gold Nanoparticles with Different Sizes and Different Capping Ligands. Colloids Surf. B Biointerfaces.

[29] Iametti S., Marengo M., Miriani M., Pagani M.A., Marti A., Bonomi F. (2013). Integrating the information from proteomic approaches: A “thiolomics” approach to assess the role of thiols in protein-based networks. Food Res. Int..

[30] Montgomery D.C. (2017). Design and Analysis of Experiments.

[31] Jimenez-Ruiz A., Perez-Tejeda P., Grueso E., Castillo P.M., Prado-Gotor R. (2015). Non-functionalized gold nanoparticles: Synthetic routes and synthesis condition dependence. Chem. Eur. J..

[32] Iwaki S., Aono S., Hayakawa K., Fu B.X., Otobe C. (2020). Changes in Protein Non-Covalent Bonds and Aggregate Size during Dough Formation. Foods.

[33] Kuktaite R., Larsson H., Johansson E. (2004). Variation in Protein Composition of Wheat Flour and Its Relationship to Dough Mixing Behavior. J. Cereal Sci..

[34] Delcour J.A., Joye I.J., Pareyt B., Wilderjans E., Brijs K., Lagrain B. (2012). Wheat Gluten Functionality as a Quality Determinant in Cereal-Based Food Products. Annu. Rev. Food Sci. Technol..

[35] Schmid M., Wieser H., Koehler P. (2016). Isolation and characterization of High-Molecular-Weight (HMW) gliadins from wheat flour. Cereal Chem..

[36] Schmid M., Wieser H., Koehler P. (2017). Disulphide structure of high-molecular-weight (HMW-) gliadins as affected by terminators. J. Cereal Sci..

[37] Ferrante P., Masci S., D’Ovidio R., Lafiandra D., Volpi C., Mattei B. (2006). A Proteomic approach to verify in vivo expression of a novel γ-gliadin containing an extra cysteine residue. Proteomics.

[38] Ferrante P., Gianibelli M.C., Larroque O., Lafiandra D., Masci S. (2008). Considerations about the effect of incorporation of two rare LMW-GS in durum wheat in comparison to bread wheat doughs. Options Mediterr..

[39] Lutz E., Wieser H., Koehler P. (2012). Identification of disulfide bonds in wheat gluten proteins by means of Mass Spectrometry/Electron Transfer Dissociation. J. Agric. Food Chem..

[40] Iwaki S., Hayakawa K., Fu B.-X., Otobe C. (2021). Changes in Hydrophobic Interactions among Gluten Proteins during Dough Formation. Processes.

[41] Aussenac T., Carceller J.L., Kleiber D. (2001). Changes in SDS Solubility of Gluten Polymers during Dough Mixing and Resting. Cereal Chem..

[42] Peng P., Wang X., Zou X., Zhang X., Hu X. (2022). Dynamic Behaviors of Protein and Starch and Interactions Associated with Glutenin Composition in Wheat Dough Matrices during Sequential Thermo-Mechanical Treatments. Food Res. Int..

[43] Abedi E., Pourmohammadi K. (2021). Chemical Modifications and Their Effects on Gluten Protein: An Extensive Review. Food Chem..

[44] Yang Y., Saand M.A., Huang L., Abdelaal W.B., Zhang J., Wu Y., Li J., Sirohi M.H., Wang F. (2021). Applications of Multi-Omics Technologies for Crop Improvement. Front. Plant Sci..

