# Supplementary material for: Gold Nanoparticles as a Possible Tool to Untangle Some Structural Features of the Gluten Network
Source: Foods. 2025 Nov 21;14(23):3985. doi: 10.3390/foods14233985 (PMC12691966; doi:10.3390/foods14233985)
Supplement: Supplementary file 1 [file foods-14-03985-s001.zip › Supplementary material.pdf]

SUPPLEMENTARY MATERIAL

Gold Nanoparticles as a Possible Tool to Untangle Some Structural Features of the Gluten Network

Davide Emide [1,†], Giovanni D'Auria [2,†], Stefania Iametti [1,*], Alberto Barbiroli [1], Mauro Marengo [3], Gianfranco Mamone [4], Pasquale Ferranti [2] and Francesco Bonomi [1]

[1] Department of Food, Environmental and Nutritional Sciences (DeFENS), Università degli Studi di Milano, Via G. Celoria 2, 20133 Milan, Italy; davide.emide@unimi.it (D.E.); alberto.barbiroli@unimi.it (A.B.); francesco.bonomi@unimi.it (F.B.)
[2] Department of Agricultural Sciences, University of Naples Federico II, 80055 Portici, Italy; giovanni.dauria@unina.it (G.D.); pasquale.ferranti@unina.it (P.F.)
[3] Department of Drug Science and Technology, University of Turin, 10125 Turin, Italy; mauro.marengo@unito.it
[4] Institute of Food Sciences, National Research Council, 83100 Avellino, Italy; gianfranco.mamone@isa.cnr.it
* Correspondence: stefania.iametti@unimi.it
† These authors contributed equally to this work.

SUPPLEMENTARY TABLES

Table S1. Description of experimental samples used for AuNP-protein binding analysis. Semolina suspensions or extracts were incubated with gold nanoparticles (AuNPs) in cold buffered saline or cold buffered 1% SDS, followed by washing with ethanol (EtOH) or ethanol/dithiothreitol (EtOH/DTT).

Sample	Source	Treatment
A	Suspension	50 mg semolina + 1 mL cold buffered saline + 30 µL AuNP, washing EtOH
B	Suspension	50 mg semolina + 1 mL cold buffered saline + 30 µL AuNP, washing EtOH/DTT
C	Extract	1 mL extract in cold buffered saline + 30 µL AuNP, washing EtOH
D	Extract	1mL extract in cold buffered saline + 30 µL AuNP, washing EtOH/DTT
E	Suspension	50 mg semolina + 1mL cold buffered 1% SDS + 30 µL AuNP, washing EtOH
F	Suspension	50 mg semolina + 1 mL cold buffered 1% SDS + 30 µL AuNP, washing EtOH/DTT
H	Extract	1 mL extract in cold buffered 1% SDS + 30 µL AuNP, washing EtOH
L	Extract	1 mL extract in cold buffered 1% SDS + 30 µL AuNP, washing EtOH/DTT

Table S2. List of proteins and peptides identified across the different experimental conditions used in this study.

Table S3. MS identification of disulfide-bonded peptides identified across the different experimental conditions used in this study.

SUPPLEMENTARY FIGURE

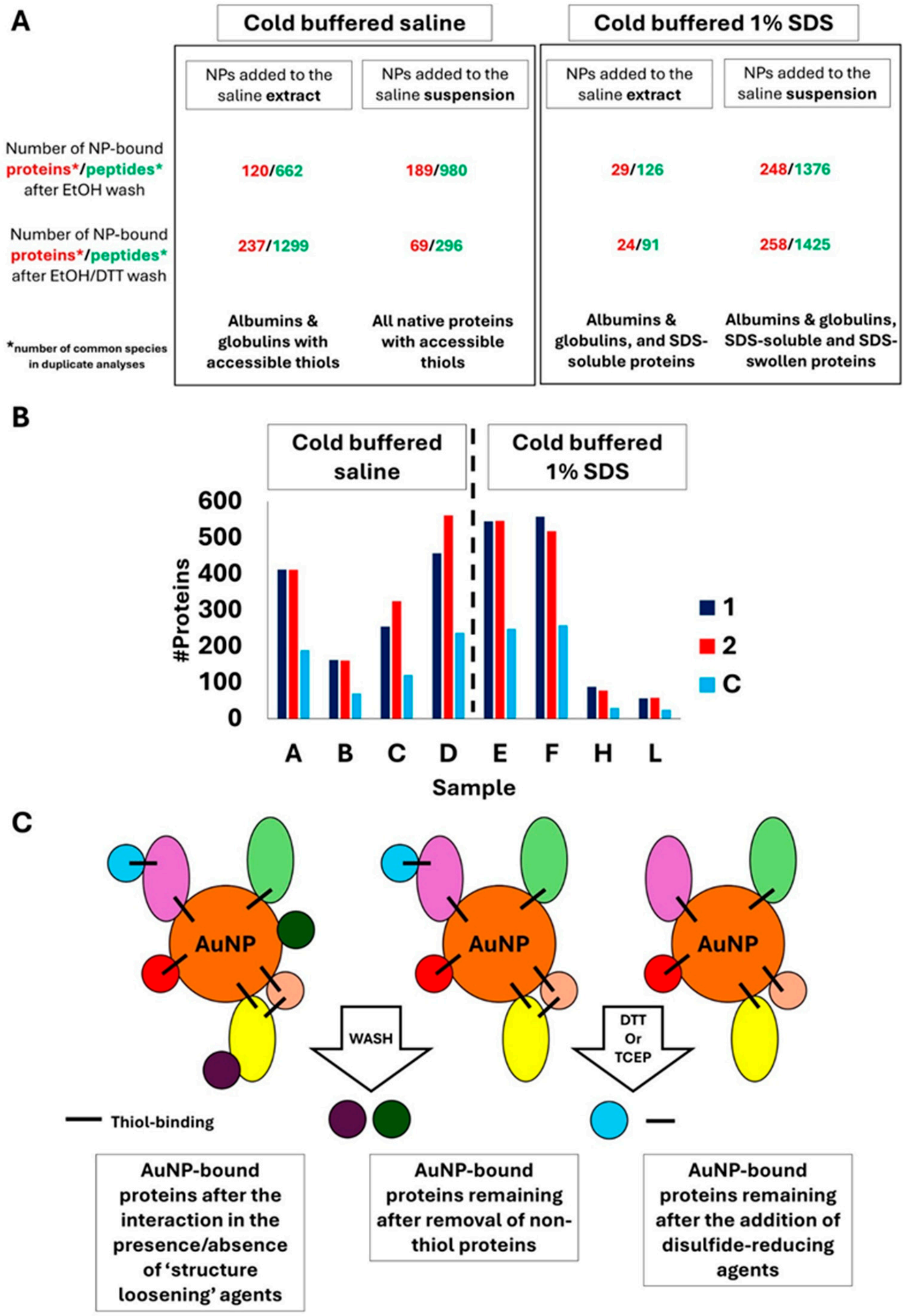

Figure S1. Characterization of nanoparticle (NP)-bound proteins under different extraction and washing conditions. (A) Number of proteins and peptides bound to gold nanoparticles (AuNPs) after sequential washing steps (EtOH or EtOH/DTT) in cold buffered saline versus cold buffered 1% SDS, comparing protein extracts and suspensions. (B) Quantification of AuNP-bound proteins identified across samples (A-L) under the two buffer conditions. Bars represent duplicate analyses and their intersection (1, 2,C). (C) Schematic representation of AuNP–protein interactions: initial binding of proteins with accessible thiols, removal of non-thiol proteins after washing, and release of disulfide-bound proteins upon treatment with reducing agents (DTT or TCEP).
